# Increasing rate of anti-SARS-CoV-2 antibodies between the first and second waves of COVID-19 in São Paulo, Brazil: A cross-sectional blood donors-based study

**DOI:** 10.1016/j.clinsp.2022.100016

**Published:** 2022-02-18

**Authors:** Nayara Moya Rios do Vale, Flavia Roche Moreira Latini, Carine Prisco Arnoni, Rafael Martins Parreira, Manoel João Batista Castelo Girão, Afonso José Pereira Cortez, Tatiana Carvalho de Souza Bonetti

**Affiliations:** a Colsan ‒ Associação Beneficente de Coleta de Sangue, São Paulo, SP, Brazil; b Departamento de Ginecologia, Universidade Federal de São Paulo, São Paulo, SP, Brazil

**Keywords:** SARS-CoV-2, Antibody, COVID-19, Seroprevalence, Blood donors, Brazil

## Abstract

**Background:**

SARS-CoV-2 infections rapidly spread along with Brazilian territory with heterogeneous transmission and mortality rates, mostly depending on region and period. Investigation of SARS-CoV-2 antibodies is an important tool to understand virus circulation. Given that blood donors are a representative casuistic of a healthy population, the authors evaluated the seroprevalence of IgG and IgM COVID-19 antibodies in 2,806 blood donors from a blood bank located in São Paulo, Brazil.

**Methods:**

Aiming to evaluate viral behavior over time, the authors selected samples from blood donors who donated in June and October 2020, and February 2021. To determine whether socio-demographic features affected the seroprevalence, the authors analyzed samples from three different regions from São Paulo (capital, metropolitan and countryside regions) and evaluated predictors as gender, age, educational level, race, and use of public transportation.

**Results:**

As expected, the authors observed that seroprevalence increased over time. Seroprevalence was greater in São Paulo city compared to metropolitan and countryside regions, being smallest in the countryside. Characteristics associated with a lower percentage of antibodies were age above 50 years, higher educational level, self-declared Caucasian, and use of individual transportation.

**Conclusion:**

In conclusion, blood donors' samples proved to accurately reflect virus circulation in the healthy population.

## Highlights

• Anti-SARS-CoV-2 antibodies in blood donors in the state of São Paulo.
• High prevalence of anti-SARS-CoV-2 antibodies in donors using public transport.

## Introduction

The first cases of SARS-CoV-2 infections were reported in China in December/2019 [1]. The virus spread rapidly worldwide, and the first case was officially reported in Brazil in February 2020. After 16-months of the first reported case, Brazil reached 500,000 deaths attributed to COVID-19. During this period, Brazil faced a dramatic political crisis that possibly contributed to increased COVID-19 transmission rates [2,3]. Incidence and mortality rates varied widely, depending on region and other demographic predictors [4,5]. Strategies adopted to control viral spread were also not consistent among different states of Brazil over time [6].

Contextualizing the worldwide situation, Brazil jumped from 11th to 3rd position in the number of cases comparing data from April 2020 to June 2021. Cases are in general considered confirmed by a positive RT-PCR test from a respiratory tract sample, which can be associated with either symptomatic or asymptomatic individual status [7].Individuals harboring the virus are frequently asymptomatic: a systematic review showed a variation in the proportion of asymptomatic individuals with positive RT-PCR from 6.3 to 96%, depending on the analyzed cohort, including several design strategies from different countries such as the United States, Germany, Argentine, England, France, Chile, Japan, India, and others [8].

The presence of antibodies is a good indicator of surveillance and can predict viral circulation [7].The presence of SARS-CoV-2 antibodies can also be associated with clinical status. Previous studies related to the presence of antibodies in asymptomatic individuals ranged from 21.7 to 85% [8]. In the Brazilian population, a previous study performed between July and August 2020, revealed that among individuals with antibodies against SARS-CoV-2, 26% were asymptomatic in a cohort of 3,156 inhabitants of different regions in the State of Maranhão, Brazil [9]. In outpatients from a large public hospital from São Paulo city, Brazil, in a period between June and August 2020, from 61 out of 439 patients with SARS-CoV-2 antibodies, 32.8% were asymptomatic [10].

Regardless of symptoms, several studies evaluated seroprevalence worldwide. A recent systematic review describes a wide range of seroprevalence among studies since viral circulation and period might have largely contributed to results [11]. Concerning Brazil, seroprevalence analysis of 25,025 and 31,165 individuals from 27 federation units during May and June 2020, respectively, showed results as low as 1% and as high as 25% depending on region evidencing that pandemic is very heterogeneous around the country [12]. In a study performed in Silva [9] and another outpatient cohort from São Paulo [10], seroprevalences were 40.4% and 13.9%, respectively. Among 4,987 healthcare workers from a public hospital in São Paulo city, antibody positivity was 14% in May 2020 [13], and 5.5% among 1,996 workers from a private hospital evaluated in June 2020 [14]. Additionally, two studies have discussed seroprevalence in blood donors from Rio de Janeiro, Manaus, and São Paulo, Brazil. Analysis of 2,857 blood 'donors' samples from Rio de Janeiro in April 2020, positivity was 3.8% [15]. Another study showed increasing seroprevalence in blood donors from São Paulo and Manaus from February to October 2020 [16].

Brazil is the biggest country in Latin America, and the first case of COVID-19 was reported on February 26th 2020, in São Paulo, the biggest and most densely populated Brazilian city. São Paulo city concentrates about 12-millions of inhabitants, which represents 26% of São Paulo state, while São Paulo state concentrates about 46 million of inhabitants, which represents 21% of the country's population. The first COVID-19 case was reported in a man one week after he returned from Italy. Less than a month later, on March 20th 2020, the state of community transmission of the coronavirus in the national territory was declared by the Ministry of Health. Additionally, on March 22nd 2020, social distancing and quarantine interventions were implemented throughout the State of São Paulo, following the WHO recommendations and comparable to European countries.

Blood 'donors' population can be an interesting sample to evaluate virus circulation since an individual must be healthy to donate blood, and COVID-19 is commonly asymptomatic [17]. To the best of our knowledge, besides Brazilian studies were performed with cohorts from Rio de Janeiro, São Paulo, and Manaus [15,16] seroprevalence investigation is still infrequent in this population 18, 19, 20, 21, 22, 23. The authors investigated the presence of SARS-CoV-2 antibodies in three different periods during the pandemic, aiming to provide information about viral circulation. Serum samples stored were randomly selected from blood donations performed in different regions of São Paulo state (capital, metropolitan, and countryside) from June and October 2020 and February 2021.

## Methods

### Setting

This study was developed by a collaboration between the Beneficent Association of Blood Collection (COLSAN) and Federal University of São Paulo – Paulista School of Medicine (UNIFESP-EPM), both located in São Paulo city. COLSAN is the largest blood collection center in São Paulo, with 11 collection sites and approximately 13,500 donations per month, representing 16% of all blood collection from the state and 4.4% of blood collection from Brazil. This study was approved by the Research Ethics Committee of UNIFESP (Project CEP / UNIFESP n°: 1487/2020).

### Study design and sampling

The authors evaluated sera samples from regular blood donors for the presence of anti-SARS-CoV-2 antibodies in three periods of the COVID-19 pandemic in São Paulo, Brazil; June/2020, October/2020, and February/2021. According to Brazilian blood bank regulations, donor samples must be stored for 6-months. A total of 2,806 samples from sera bank were included in the study. The subject's selection was based on data obtained from a questionnaire filled up at the moment of blood donation, which was available in the electronic system of the institution. After the authors had defined the three periods to be evaluated, samples were randomly included in the study according to the date of blood donation: June/2020 (926 samples), October/2020 (940 samples), and February/2021 (940 samples). It is important to highlight that the COVID-19 vaccination campaign in São Paulo started on January 19^th^, 2021, for health professionals. Hence, only in the February 2021 samples, the subjects could have received the vaccine. A total of 10 samples of the February group were not eligible for this study due to previous vaccination.

Since the beginning of the COVID-19 pandemic, Brazilian blood bank regulations were updated, and all blood donors must have been free of respiratory symptoms for at least 30 days before the donation. Therefore, there was no subject with recent symptoms associated with SARS-COV-2 infection, confirmed or not, involved in the study. All donors signed the informed consent form for blood donation as routine and answered a questionnaire including information about previous COVID-19 tests, the presence of symptoms and risk factors for SARS-CoV-2 infection, as well as information about relevant contacts and type of transportation used. As a consequence, all subjects were healthy at the time of donation and had no reported health issues in the 4-weeks before donation.

### Anti-SARS-CoV-2 antibody detection

The detection of SARS-CoV-2 antibodies was carried out using the Elecsys® Anti-SARS-CoV-2 kit (antibodies IgG and IgM) (Roche Diagnostics, Mannheim, Germany). Briefly, it is an automated sandwich, double-antigen electrochemiluminescent immunoassay that employs recombinant protein representing the nucleocapsid antigen of the virus, using Cobas e801 equipment following the manufacturer's instructions. As indicated by the manufacturer, samples with a DO/Cut-Off result >1.0 were considered positive. The validation and performance of the kit are well-established 24, 25, 26. Elecsys® Anti-SARS-CoV-2 kit detects total antibodies and does not differentiate the antibody classes (IgG, IgM, or IgA).

### Data collection and analysis

Subject data (age, gender, race, occupation, educational level, place of blood donation) were obtained from blood donors' electronic records. The information was compiled in Microsoft Office Excel (Microsoft Corporation, USA) spreadsheet and analyzed using JASP (version 0.14.1.0). Categorical variables were described as frequency and percentages. Continuous variables were described by their means and standard deviations. Univariate and multivariate logistic regression models were performed to evaluate the association of demografic variables with the presence of antibody. The authors used independent group *t*-tests or ANOVA for continuous variable comparisons and Qui-Square tests for categorical ones accordingly. For all analyses, the authors used SPSS 21 software (IBM Software), and p-values less than 0.05 were considered statistically significant.

## Results

### Demographic characteristics of blood donors

The authors analyzed 2,806 sera samples from blood donors between 16 and 69 years old (37.4 ± 12.0), from both male and female sex, collected on four blood donation sites, two in São Paulo capital (Campo Limpo and Tatuapé blood donation sites), one in the metropolitan region of São Paulo (São Bernardo do Campo blood donation site) and one in a countryside city (Sorocaba blood donation site). Most subjects were Caucasian and had high school degrees. Demographic characteristics of the population in the three collection periods according to SARS-CoV-2 seroprevalence are described in Table 1.

**Table 1:** General characteristics of subjects included in the study (total) and split according to three-time groups of the study.

**Period of blood collection**	**Total**	**June 2020**	**October 2020**	**February 2021**	**p**
n	2806	926	940	940	
Blood collection site					
Capital	1407 (50.1%)	447 (48.3%)	480 (51.1%)	480 (51.1%)	0.516
Metropolitan region	715 (25.5%)	255 (27.5%)	230 (24.5%)	230 (24.5%)	
Countryside	684 (24.4%)	224 (24.2%)	230 (24.5%)	230 (24.5%)	
Age groups					
16‒29	827 (29.5%)	279 (30.1%)	277 (29.5%)	271 (28.8%)	0.702
30‒39	774 (27.6%)	261 (28.2%)	268 (28.5%)	245 (26.1%)	
40‒49	686 (24.4%)	214 (23.1%)	224 (23.8%)	248 (26.4%)	
≥50	519 (18.5%)	172 (18.6%)	171 (18.2%)	176 (18.7%)	
Age – mean±SD (range)	37.4±12.0 (16–69)	37.2±12.0 (16–69)	37.3±11.9 (16–69)	37.9±12.2 (16–69)	0.415
Gender					
Female	1314 (46.8%)	430 (46.4%)	459 (48.8%)	425 (45.2%)	0.279
Male	1492 (53.2%)	496 (53.6%)	481 (51.2%)	515 (54.8%)	
Ethnic					
Asian	30 (1.1%)	14 (1.5%)	9 (1.0%)	7 (0.7%)	0.090
Caucasian	2505 (89.6%)	813 (87.8%)	856 (91.4%)	836 (89.5%)	
Afro-descendants	262 (9.4%)	99 (10.7%)	72 (7.7%)	91 (9.7%)	
School grade					
Elementary school	220 (7.8%)	80 (8.6%)	62 (6.6%)	78 (8.3%)	<0.001
High school	1634 (58.3%)	476 (51.4%)	594 (63.2%)	564 (60.1%)	
University education	950 (33.9%)	370 (40.0%)	284 (30.2%)	296 (31.6%)	

### Seroprevalence by region

**Fig. 1:**
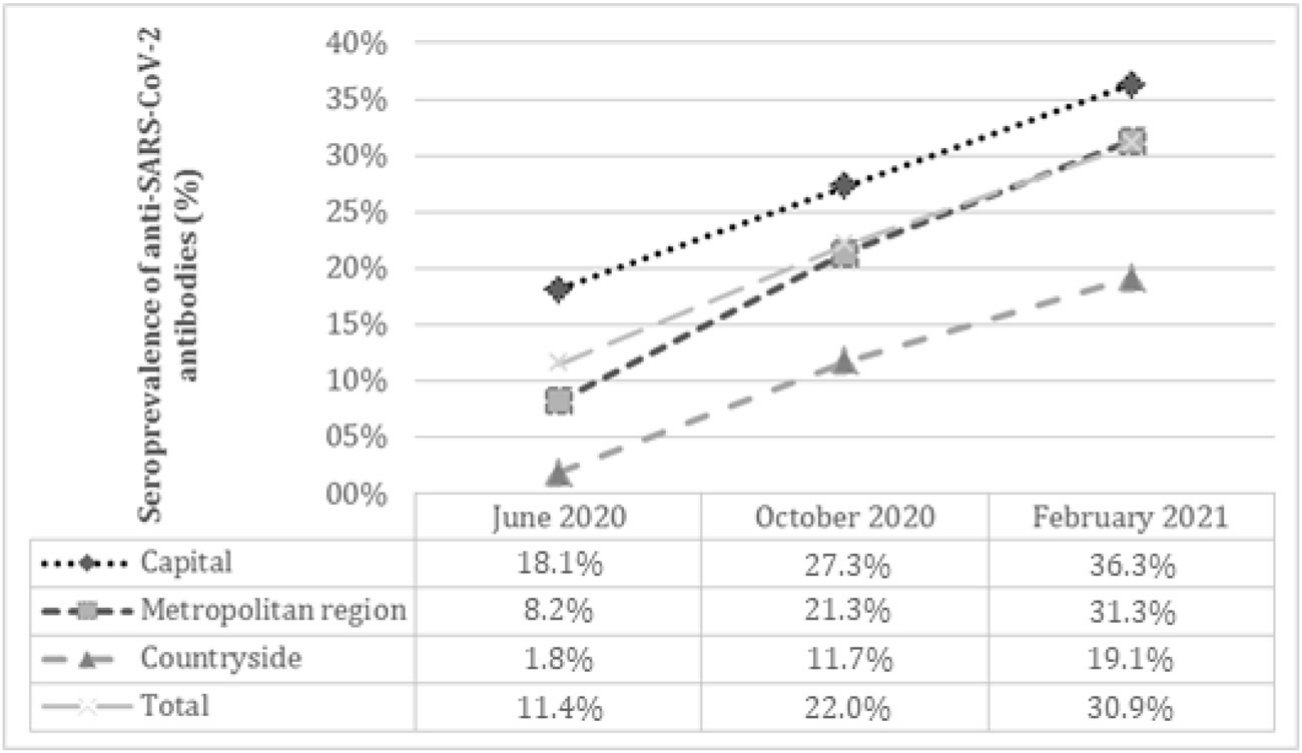
Univariate analysis of the seroprevalence of anti-SARS-CoV-2 antibodies in the three periods evaluated and according to the blood donation sites. The seroprevalence for the whole group and according to the blood donation sites increased over time, and comparisons by qui-square test showed statistical significance (*p* < 0.001).

The overall seroprevalence was 21.5%, considering three periods of blood donation. In June 2020, when Brazil faced the first COVID-19 wave, the average was 5,722 cases and 238 deaths per day in the State of São Paulo. Overall seroprevalence was 11.4%, which varied by geographic region. The seroprevalence in the capital was significantly higher (18.1%) than the others: it decreased as the blood donation site moved away from the capital, being 8.2% and 1.8% in metropolitan and countryside regions, respectively (*p* < 0.001). In October 2020, the pandemic in Brazil was under relative control, with an average of 4,209 cases and 119 deaths per day in the São Paulo State. In February 2021, Brazil faced the second wave of COVID-19 pandemic, when 9,437 cases and 230 deaths per day were reported. The seroprevalence had a significant increase from June 2020 to October 2020 and from October 2020 to February 2021 in the three regions analyzed (Fig. 1). It is interesting to note that seroprevalence profiles were maintained in three periods evaluated where capital was higher than metropolitan region and country.

### Seroprevalence by demographic characteristics of blood donors

**Fig. 2:**
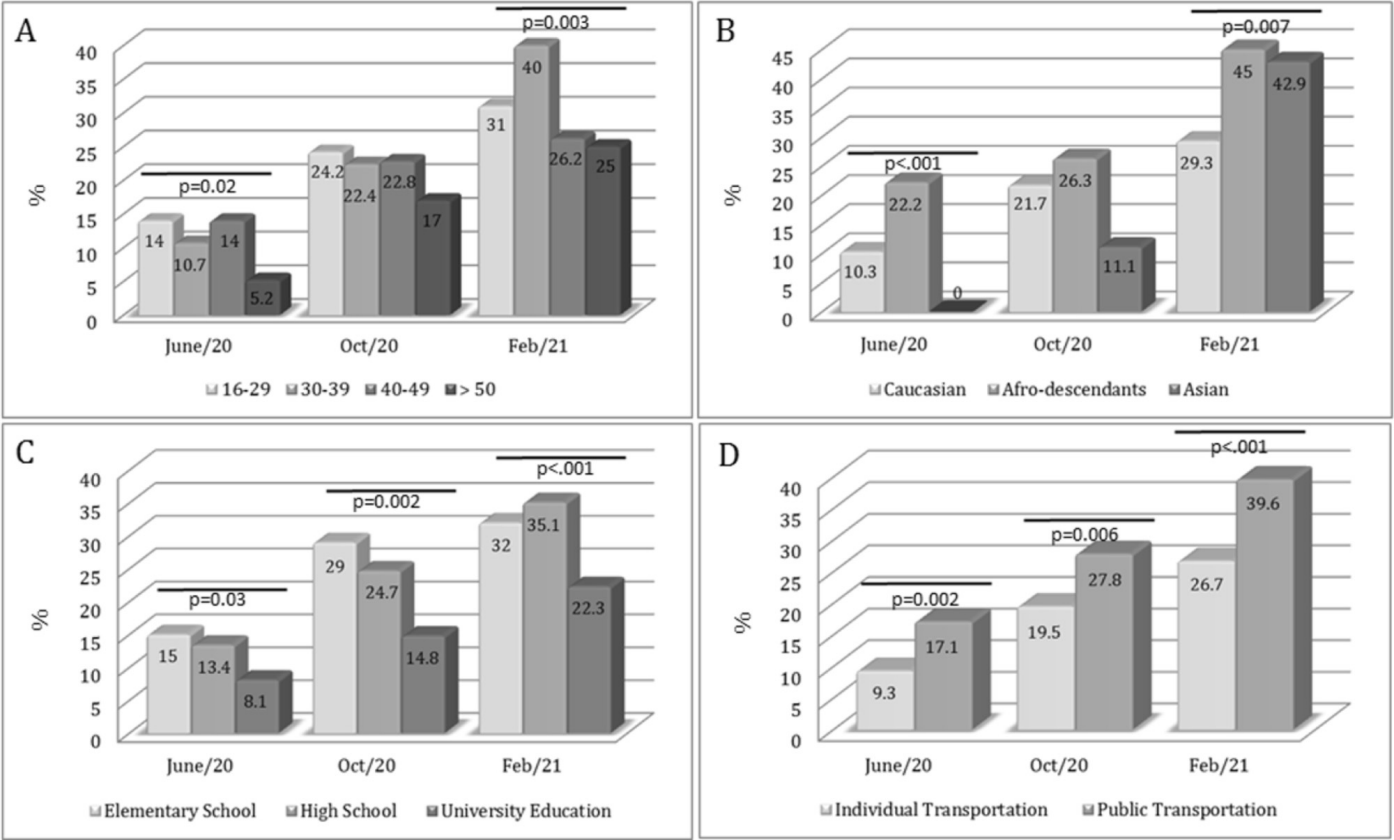
Percentage of seroprevalence of anti-SARS-CoV-2 antibodies in the three periods evaluated in accordance with the age groups (A), race (B), school grade (C) and use of public transportation (D). The comparisons among seroprevalences over the categories were performed by Qui-Square test and those with significant differences are shown by lines above the bars with the respective p-values.

The seroprevalence of antibodies to SARS-CoV-2 was not different for male and female sex in general (22.6% vs. 20.2%; *p* = 0.144), or in each period of blood donation separately, as June 2020 (12.1% vs. 10.7%; *p* = 0.573), October 2020 (23.9% vs. 20.0%; *p* = 0.177) or February 2021 (31.5% vs. 30.1%; *p* = 0.710). On the other hand, the seroprevalence of antibodies to SARS-CoV-2 was lower in population above 50-years old in the first period of this study (June 2020) but showed a gradual increase over the months, reaching 25% of positivity in February 2021, getting a similar rate to other ages (Fig. 2A). The seroprevalence was higher among Afro-descendants than in the Caucasian population in all analyzed periods. Despite the data showing an increase of seroprevalence in the Asian population in February 2021, it should not be considered that just seven blood donors compound this group (Fig. 2B). The school grade was also determinant given that there was an inverse relation of school grade and seroprevalence of antibodies to SARS-CoV-2 in all analyzed periods (Fig. 2C).

Public transportation is considered one of the main risk factors for population contamination to SARS-CoV-2. The authors evaluated the seroprevalence of anti-SARS-CoV-2 antibodies according to the use of public transportation by blood donors, showing a significantly higher seroprevalence among those who use public (29.2%) compared to individual transportation (18.9%; p = 0.003). This profile was maintained in the three periods evaluated (Fig. 2D).

The univariate and multivariate logistic regression models were performed to confirm the association between demographic data and the presence of anti-SARS-CoV-2 antibodies. The period of analysis, age range, race, school grade, and type of transportation predicts the presence of anti-SARS-CoV-2 antibodies in both univariate and multivariate analysis (adjusted for confounders) (Table 2).

**Table 2:** Univariate and multivariate logistic regression analysis to predict the presence of anti-SARS-CoV-2.

	**Univariated regression**		**Multivariated regression**	
	**Odds Ratio (95% CI)**	**p**	**Odds Ratio (95% CI)**	**p**
**Period**				
June 2020	Reference	‒		
October 2020	2.185 (1.694‒2.817)	<0.001	2.151 (1.631‒2.838)	<0.001
February 2021	3.451 (2.701‒4.410)	<0001	3.440 (2.633‒4.496)	<0.001
**Age groups**				
16‒29	Reference	‒		
30‒39	1.053(0.836‒1.327)	0.662	1.093 (0.852‒1.402)	0.485
40‒49	0.906 (0.71‒1.157)	0.431	0.849 (0.652‒1.105)	0.223
>50	0.629 (0.473‒0.838)	0.002	0.543 (0.395‒0.745)	<0.001
**Ethnic**				
Caucasian/Asian	Reference	‒		
Afro-descendants	1.770 (1.340‒2.338)	<0.001	1.594 (1.174‒2.165)	0.003
**School grade**				
Elementary school	Reference	‒		
High school	1.002 (0.724‒1.386)	0.992	0.865 (0.6000‒1.247)	0.437
University education	0.510 (0.358‒0.727)	<0.001	0.472 (0.315‒0.706)	<0.001
**Transportation**				
Individual	Reference	‒		
Public	1.767 (1.454‒2.148)	<0.001	1.558 (1.267‒1.917)	<0.001

## Discussion

The authors randomly selected blood ' 'donor's sera samples from donations performed in June and October 2020 and February 2021 to characterize the seroprevalence of antibodies against COVID-19 in different regions of the São Paulo state. As viral circulation demonstrated to be very heterogeneous depending on region and period, the authors selected donors from two different blood collection sites from São Paulo city (Campo Limpo and Tatuapé), from a city from the metropolitan region of São Paulo (São Bernardo do Campo) and also from a country city of São Paulo (Sorocaba). The blood 'donors' samples included in this study are representative of the general blood donors in the institution, as they present similar demographic characteristics of gender, ethnicity, school grade and age (data not shown).

Analyzing IgG and IgM (Elecsys® Anti-SARS-CoV-2 kit, Roche) antibodies in 2,806 samples, the authors observed an increase in overall seroprevalence along time, from 11.4% in June 2020 to 22.0% on October 2020, reaching 30.9% in February 2021. Periods of selected donations were defined to include the COVID-19 first wave in June 2020, when the authors expected that virus circulation was already significant. Thus, the authors decided to take a snapshot after 4-months, when the pandemic was relatively under control in São Paulo state in October 2020. Moreover, with another gate of 4-months, the authors evaluated antibodies prevalence in February 2021, before mass administration of COVID-19 vaccines, excluding a few cases when a blood donor had already received the first dose. These results corroborate epidemiologic data that shows an increase in the number of cases one year after the first case was reported in February 2020. A similar analysis was performed in Stockholm when 2,600 blood donors were serologically tested in June, and November 2020, as well as in February 2021, and positivity was present in 8.3%, 14.1%, and 19.2%, respectively [22]. Although political strategies adopted to control pandemic and population features were completely distinct, authors also observed an increase in the presence of antibodies in blood donors over time. It is important to highlight that people with suspected or confirmed infection by SARS-CoV-2 in the last 30-days could not follow with blood donation due to blood bank regulations. This condition implies that the analysis from donors who could bear antibodies anti-SARS-CoV-2 due to recent infection was not included. Then, the seroprevalence observed in the present study's findings can be higher once the possible recent infected individuals were not included. Moreover, 117 subjects reported previous SARS-CoV-2 infection confirmed by PCR occurred 30 or more days before blood donation. From those, almost all (n = 102, 87%) were antibodies positive (data not shown). This information suggests that most of the blood donors evaluated in this study (83%) probably were exposed to the virus but had no symptoms, which corroborate with the literature about the proportion of asymptomatic infections caused by SARS-CoV-2 [17].

Collection time was the most important predictor associated with seroprevalence. Therefore, to allow any comparison, it is crucial that the presence of antibodies always have to be contextualized to period. Regarding blood donors from São Paulo city, a previous study presented a seroprevalence lower than 1% in February and March 2020, reaching 13.6% in June 2020 [16]. Considering the use of a different antibody detection kit and the fact that São Paulo city is very heterogeneous across districts, the authors might risk suggesting that similar results were observed in June 2020, as the authors detected 18.1% seroprevalence analyzing samples from the south (Campo Limpo) and east (Tatuapé) regions of the city.

Stratifying analyzed donor samples according to sites where donations were performed, as donors regularly donate on the neighborhood of their living or working addresses, the authors could observe the reflection of region situation on the study's casuistic. Seroprevalence at São Paulo city was the highest among blood donors during the three evaluated periods. As São Paulo city, the largest city of Brazil, represents 26% of the population from all 645 cities from São Paulo state, the expected higher degree of social interaction might proportionally increase viral circulation [4]. As larger cities present higher transmission rates, smaller cities present a delay, showing an increase in the long-term course of the pandemic [4]. It was exactly what the authors observed in the countryside, Sorocaba. While São Paulo city seroprevalence started with 18.1% in June 2020, it was 1.8% in Sorocaba, clearly demonstrating that viral circulation was still mostly centralized. After the initial spread, in February 2021, seroprevalence in São Paulo city was nearly 2-times greater, 36.3%, compared to June 2020, while it was approximately 10-times superior, 19.1%, in the countryside. The authors observe, as expected, that incidence rates tended to decrease in large cities and to increase in small cities along with the surge [4]. Evaluating the metropolitan region of São Paulo, the authors found an intermediate situation. As São Bernardo do Campo is a larger city compared to Sorocaba and is geographically located close to São Paulo city, seroprevalence started with 8.2% in June 2020 and reached 31.3% in February 2021, a percentage almost 4-times higher.

Similar to available data on literature, the authors did not observe differences in the presence of antibodies between males and female blood donors, as other authors also demonstrated in blood donors from Rio de Janeiro, Brazil [15], the Netherlands [20], Denmark [18] and Saudi Arabia [23].

In the analyzed cohort, the population > 50 years old had the lower seroprevalence in June 2020, which is in line with older individuals expected to have mostly restricted social interaction, given that age is the main risk factor to COVID-19 complications [27,28]. However, in the following periods, the seroprevalence in > 50 years old blood donors was similar to other groups of ages (16 to 29, 30 to 39, and 40 to 49), showing the difference between seroprevalence on donors under 50 and above 50 years have decreased over time. This observation probably reflects the adhesion of restrictive social measures, which was more restrictive on the elderly population during the first months of the pandemic but was weakening month after month. Another study with blood donors in Rio de Janeiro, Brazil, showed that donations from April 2020 presented 5.2%, 3.7%, and 2.9% seroprevalence on donors with 18‒29, 30‒49, and 50‒69, respectively [22].

Several aspects corroborate to impaired adhesion of social distancing measures by the low-incoming working class: living in highly populated areas (“*favelas*”), precarious hygiene and sanitization conditions, lack of remote work as an alternative and overcrowded public transportation [29,30]. The present study's results reflect the above-mentioned historical background. Self-declared Black, lower school grades, and use of public transportation were related to higher seroprevalence.

Race influences on COVID-19 seropositivity have already been described in a large national survey. From 25,025 (May 2020) and 31,165 (June 2020) tested individuals from all Brazil federal units, comparison between Caucasian, Afro-descendants, or mixed population showed that prevalence of antibodies on Afro-descendants and mixed population were almost 5-times higher in both analyzed periods [12]. Furthermore, even though high socioeconomic individuals who traveled abroad first brought COVID-19 to Brazil, hospitalization and deaths of Afro-descendant individuals rapidly reached and overcame those from Caucasians [29]. Agreeing with the literature, in all three periods analyzed, the authors observed a higher percentage of antibodies positivity in Afro-descendants compared to Caucasians. In February 2021, 45% of self-declared Afro-descendant donors presented COVID-19 antibodies.

Additionally, the lack of an alternative to the use of public transportation to decrease social interaction during a pandemic is another challenge to a less favored population. As the authors observed, a previous study that evaluated the presence of SARS-CoV-2 antibodies in health care workers in São Paulo associated the use of public transportation with increased seroprevalence [14]. The authors demonstrated that the presence of antibodies was around 50% higher in all three periods when public transportation instead of individual transportation was used. Moreover, the authors observed that educational level was associated with seroprevalence as higher grades were inversely related to antibodies positivity. Other authors also reported lower school grades to higher positivity in Rio de Janeiro blood ' 'donor's cohort, as it was 4.7% in individuals with no university education and 2.8% in individuals with university education [15].

At last, the authors analyzed all demographic data in multivariate logistic regression analysis and confirmed the association of them as predictors for the presence of anti-SARS-CoV-2 antibodies. Summarizing, data here provided show that the blood ' 'donor's population is a well representative cohort. Even though the presence of symptoms became part of clinical screening during the blood ' 'donor's interview, it cannot be used as an indication of viral circulation since it is well known that asymptomatic cases are common [17]]. Therefore, a good indicator is the presence of antibodies.

Thus, investigation of seroprevalence in blood donors from different regions across São Paulo state was an effective strategy to enlighten viral behavior. As a consequence of political chaos, adhesion to restrictive social measures was not consistent, and investigation of antibodies in blood donors reflected heterogeneity of seroprevalence in specific groups. However, the correlation of SARS-CoV-2 antibodies with socio-demographic features could hint at information about vulnerable populations and drive political strategies to combat the pandemic.

## CRediT authorship contribution statement

**Nayara Moya Rios do Vale:** Visualization, Data curation, Formal analysis, Writing – original draft. **Flavia Roche Moreira Latini:** Visualization, Formal analysis, Methodology, Writing – original draft, Writing – review & editing. **Carine Prisco Arnoni:** Visualization, Data curation, Formal analysis. **Rafael Martins Parreira:** Investigation, Funding acquisition. **Manoel João Batista Castelo Girão:** Writing – review & editing. **Afonso José Pereira Cortez:** Writing – review & editing. **Tatiana Carvalho de Souza Bonetti:** Visualization, Formal analysis, Methodology, Writing – original draft, Writing – review & editing.

## Conflicts of interest

The authors declare no conflicts of interest.
